# Keeping goblet cells unstressed: new insights into a general principle

**DOI:** 10.1038/s44318-024-00041-4

**Published:** 2024-02-02

**Authors:** Anne Bertolotti

**Affiliations:** MRC Laboratory of Molecular Biology, Francis Crick Avenue, Cambridge, CB2 0QH UK

**Keywords:** Digestive System, Translation & Protein Quality

## Abstract

Two new studies reveal how the unfolded protein response is regulated in mucin-secreting gut epithelial cells.

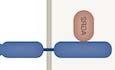

## Teaser

The regulation of unfolded protein response (UPR) sensors has been studied for over 20 years, but IRE1β has remained a mystery. IRE1β is highly expressed in goblet cells: specialized epithelial cells that secrete mucus. Two articles in this issue show that IRE1β is held in an inactive state by the goblet cell-specific chaperone AGR2; upon dissociation of AGR2, IRE1β dimerizes and becomes active. This mechanism is reminiscent of the dynamic control by BiP of three other UPR sensors: IRE1α, PERK and ATF6.

Most proteins must be folded properly to be functional and cells have evolved a series of mechanisms to ensure this happens. For proteins of the secretory pathway, this folding, assembly and modification work occurs in the endoplasmic reticulum (ER); accordingly, across different cell types, the ER has been shaped by each specific cell’s biosynthetic demands and its abundance has come to vary considerably. For example, red blood cells have no ER, whilst secretory cells, such as plasma cells, pancreatic β cells and goblet cells, have abundant ER.

Cells with a high secretory capacity need tight quality control mechanisms to provide sufficient folding capacity in the ER. This is ensured by the UPR, which in mammals is composed of a set of coordinated homeostatic pathways orchestrated by three transmembrane UPR sensors. These ER proteins initiate signalling cascades that work by both reducing the rate of protein synthesis and increasing the amount of ER-resident chaperones. The first of these UPR sensors, IRE1, was discovered in yeast; by homology two IREs were later cloned in mammals (Tirasophon et al, 1998; Wang et al, 1998). IRE1α is expressed broadly whilst IRE1β was found in the gut epithelium (Bertolotti et al, 2001). The effector domain of IRE1 contains a kinase and an endonuclease domain; when activated, it cleaves a specific mRNA (HAC1 in yeast, XBP1 in mammals) allowing its translation into a transcription factor that controls the expression of ER-resident chaperones. The second UPR sensor elicits a fast response to folding challenges in the ER, PEK/PERK, has a luminal domain homologous to IRE1 and its effector kinase domain phosphorylates the translation initiation factor eIF2α, slowing down translation (Shi et al, 1998; Harding et al, 1999). The third UPR sensor, ATF6, was discovered after the realization that in mammals, the IRE1- XBP1 branch of the UPR is not sufficient for transcriptional induction of genes encoding ER chaperones, but that it also requires two paralogues of ATF6 (Haze et al, 2001).

Knockout studies in mice revealed the physiological relevance of the UPR. PERK knockout mice die shortly after birth with a loss of pancreatic β cells, meaning that they cannot maintain glucose homeostasis (Harding et al, 2001). Although PERK is expressed in all cells, its function appears to be essential in pancreatic β cells, probably because of the high demands on the controlled production of insulin. Expression of IRE1β in the epithelial cells of the intestinal tract also suggested a specialized role, which was revealed with increased sensitivity of the *Ire1β* knockout mice to experimentally induced colitis (Bertolotti et al, 2001). Deletion of *Xbp1* also increased the susceptibility of mice to induced colitis (Kaser et al, 2008).

The effector functions of the three branches of the UPR are relatively well understood and the sensing mechanisms have been studied for over 20 years. Soon after the identification of mammalian IRE1s and PERK, antibodies were generated to conduct unbiased searches for regulators by immunoprecipitation of the endogenous proteins from cell lines and mouse tissues (Bertolotti et al, 2000). This revealed that IRE1α, β, and PERK are monomeric and inactive when bound to the ER chaperone BiP (Bertolotti et al, 2000). Perturbation of proteostasis in the ER causes the dissociation of BiP, enabling activation by dimerization or oligomerization of the stress sensors (Bertolotti et al, 2000). Although this mechanism is conserved between the three mammalian UPR sensors, an alternative model positing that IRE1 is activated by direct binding to misfolded proteins was preferred for several years.

Whilst much is known about the UPR, the framework under which it was studied did not predict all aspects; for example, two articles in this issue reveal a new component regulating IRE1β. There were some clues to suggest that IRE1β was not just another IRE1 in the gut. Now, Cloots et al, (2024) elucidate the regulation of IRE1β by taking advantage of its non-physiological toxic properties (Wang et al, 1998) when expressed in non-gut cells. The authors compared IRE1β activities in lung epithelial cells and colon goblet cells, after knocking out IRE1α. They found that overexpression of IRE1β is toxic in the lung, as was reported earlier for fibroblasts (Tirasophon et al, 1998; Wang et al, 1998), but not in the colon. This led to the suspicion that colon cells expressed an endogenous suppressor of the toxic effects of IRE1β. To explore this idea further, the authors immunoprecipitated IRE1α and IRE1β from colon cells. In addition to BiP, the previously known repressor of IRE1α and IRE1β, they found that IRE1β, but not IRE1α, interacted with the goblet-cell-specific ER chaperone AGR2 (Cloots et al, 2024). AGR2 is a protein disulfide isomerase (PDI) that is highly and uniquely expressed in goblet cells, consolidating the idea that the absence of AGR2 might explain the toxicity of IRE1β in non-goblet cells. Indeed, expression of AGR2 suppressed activity and toxicity of IRE1β in heterologous cells. Mechanistically, this occurs because AGR2 binding prevents IRE1β dimerization, in a manner similar to BiP and IRE1α (Bertolotti et al, 2000). Underscoring the pathophysiological importance of their findings, Cloots et al found that a homozygous H117Y variant in AGR2, previously identified as a cause of severe early-onset inflammatory bowel disease (Al-Shaibi et al, 2021) was unable to interact with IRE1β. AGR2 variants were also identified in disorder resembling cystic fibrosis (Bertoli-Avella et al, 2022), raising the possibility that the mechanism identified here in colon goblet cells might be relevant in the lung as well. Supporting this idea, IRE1β is also expressed in airway mucous cells (Martino et al, 2013).

In a parallel study, also published in this issue, Neidhardt et al (2024) studied the activation mechanism of IRE1β by creating an IRE1β/α chimera in Chinese hamster ovary cells. They also showed that the luminal domain of IRE1β was toxic to these cells, and that this activity could be repressed by AGR2. Reconstitution of the interaction between luminal domains of AGR2 and IRE1β with recombinant proteins showed that the domains form a one-to-one complex that is able to prevent dimerization of IRE1β/α. Although AGR2 is a PDI, the interaction between IRE1β and AGR2 does not involve disulphide bonds.

The major function of goblet cells is to synthesize and secrete mucus. Mucins are proteins of more than 5,000 amino acids and their production is highly demanding on the ER, not only because of the load, but also because of the formation of multiple disulfide bonds and oligomerization. Neidhardt et al go on to show that overexpression of MUC2 in non-goblet cells burdens the ER and derepresses AGR2-IRE1β, also overexpressed in these cells. It will be interesting to investigate whether this can be reconstituted with recombinant proteins.

**Figure 1:**
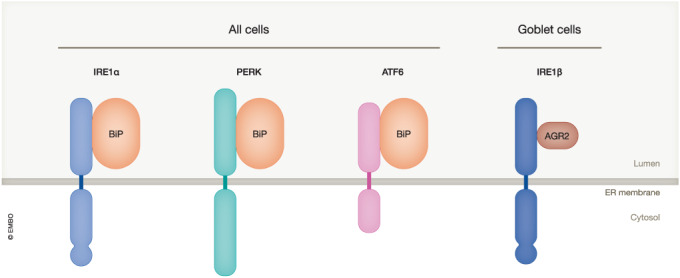
Sensors of the unfolded protein response and their regulators.

These two new studies highlight a specialized function for IRE1β in mucin goblet cells and provide an additional example of convergent evolution in the mechanisms by which ER stress sensors detect proteostatic perturbations in the ER lumen. IRE1α, PERK, and ATF6 are repressed when bound to BiP, which dissociates upon stress to allow their activation. With IRE1β, goblet cells possess an additional branch of the UPR, which is repressed by AGR2, a cell-specific chaperone, through a mechanism analogous to the repression of IRE1α by BiP (Fig. 1).

The mechanism by which ER-stress sensors are depressed has been debated. A perhaps intuitive model proposes that dissociation of the BiP-IRE complex is achieved by competition with misfolded proteins (Amin-Wetzel et al, 2017). However, in cells, BiP is present in huge excess when compared to stress sensors. This fact argues against dissociation by a competitive mechanism. This model also implies that the UPR could only be turned on after BiP is titrated out by binding to misfolded proteins. In such a scenario, high amounts of unfolded proteins would be a prerequisite for activation: this is incompatible with the homeostatic function of the UPR that requires activation by minute amounts of misfolded proteins. The situation is different for IRE1β and AGR2 because both proteins are abundant in goblet cells.

It must be noted that it is difficult to study UPR sensing using recombinant proteins. This is because IRE1 can bind to the ER chaperones in two ways: (1) as a properly folded signaling molecule, or (2) as a misfolded or unfolded client. It is difficult to ascertain that recombinant proteins are correctly folded and whether they bind BiP as a typical misfolded client or as a signaling molecule. In line with the notion that IRE1 can interact with BiP as a misfolded client and in a different way as a signaling, stress-sensing molecule, it has been reported that BiP can bind in the typical chaperone-client way (Amin-Wetzel et al, 2017) or in an atypical way, through its ATPase domain (Carrara et al, 2015). These two modes of interactions between BiP and IRE1 may represent a different folding status of the recombinant proteins. The atypical interaction spares BiP’s substrate-binding domain for binding misfolded proteins, enabling its dissociation from IRE1 and PERK through a conformational change (Carrara et al, 2015). It would be interesting to investigate which mechanism is involved in disrupting the IRE1β-AGR2 complex, bearing in mind the difficulties outlined above in producing recombinant proteins in their biologically relevant fold.

Both papers in this issue reveal cell-specific features of the UPR in mammals and raise the possibility that additional cell-specific aspects may remain to be discovered. Goblet cells offer a neat system for further studies.
